# Protection Reduces Loss of Natural Land-Cover at Sites of Conservation Importance across Africa

**DOI:** 10.1371/journal.pone.0065370

**Published:** 2013-05-29

**Authors:** Alison E. Beresford, George W. Eshiamwata, Paul F. Donald, Andrew Balmford, Bastian Bertzky, Andreas B. Brink, Lincoln D. C. Fishpool, Philippe Mayaux, Ben Phalan, Dario Simonetti, Graeme M. Buchanan

**Affiliations:** 1 Conservation Science, The Royal Society for the Protection of Birds, Edinburgh, United Kingdom; 2 Africa Partnership Secretariat, BirdLife International, Nairobi, Kenya; 3 Conservation Science, The Royal Society for the Protection of Birds, Sandy, Bedfordshire, United Kingdom; 4 Department of Zoology, University of Cambridge, Cambridge, United Kingdom; 5 United Nations Environment Programme World Conservation Monitoring Centre, Cambridge, United Kingdom; 6 Institute for Environment and Sustainability, Joint Research Centre of the European Commission, Ispra, Italy; 7 BirdLife International, Cambridge, United Kingdom; 8 Reggiani SpA, Institute for Environment and Sustainability, Joint Research Centre of the European Commission, Ispra, Italy; NASA Jet Propulsion Laboratory, United States of America

## Abstract

There is an emerging consensus that protected areas are key in reducing adverse land-cover change, but their efficacy remains difficult to quantify. Many previous assessments of protected area effectiveness have compared changes between sets of protected and unprotected sites that differ systematically in other potentially confounding respects (e.g. altitude, accessibility), have considered only forest loss or changes at single sites, or have analysed changes derived from land-cover data of low spatial resolution. We assessed the effectiveness of protection in reducing land-cover change in Important Bird Areas (IBAs) across Africa using a dedicated visual interpretation of higher resolution satellite imagery. We compared rates of change in natural land-cover over a c. 20-year period from around 1990 at a large number of points across 45 protected IBAs to those from 48 unprotected IBAs. A matching algorithm was used to select sample points to control for potentially confounding differences between protected and unprotected IBAs. The rate of loss of natural land-cover at sample points within protected IBAs was just 42% of that at matched points in unprotected IBAs. Conversion was especially marked in forests, but protection reduced rates of forest loss by a similar relative amount. Rates of conversion increased from the centre to the edges of both protected and unprotected IBAs, but rates of loss in 20-km buffer zones surrounding protected IBAs and unprotected IBAs were similar, with no evidence of displacement of conversion from within protected areas to their immediate surrounds (leakage).

## Introduction

Protected areas (PAs) form a central pillar in efforts to combat biodiversity loss and absorb a high proportion of global conservation investment [1], [2]. In 2010, the Parties of the Convention on Biological Diversity (CBD) agreed to increase total coverage of land in PAs from current levels of c. 13% [3] to 17% by 2020, targeting expansion to areas of recognised biodiversity importance, [4], which should improve the currently poor capture by PAs of the ranges of globally threatened species [5]. A recent estimate suggests that protecting the world’s most important sites for biodiversity and meeting the target of 17% coverage might cost in the region of $76 billion annually if the target is to be met by 2020, requiring a substantial increase in current levels of spending [6].

If investment in site protection is to be enhanced, the effectiveness of PAs in reducing deleterious land-cover change needs to be quantified [7]. Several recent studies suggest that PAs are effective at reducing rates of land-cover change within their boundaries [8], [9], [10], [11], [12], but have considered only deforestation. Few have looked at change across all land-cover types (an exception being the coarse-resolution global assessment of Joppa & Pfaff [13]), and none has compared changes across protected and unprotected sites of similarly recognised biodiversity value. Consequently, the effectiveness of PAs at preventing conversion of all habitats in sites of high importance remains poorly quantified.

Although several approaches have been proposed for assessing the effectiveness of PAs [14], [15], [16], [17], [18], [19], it remains difficult for several reasons, including their large number and area, limited monitoring resources, and methodological constraints [10], [20], [21], [22]. Assessments based on remotely sensed data have used measures of forest fires [10], [23], deforestation [8], [12], [24] or other land-cover changes [25], comparing such rates before and after protection, between protected and unprotected sites, or within and outside PAs. Comparing rates of change in PAs with those in unprotected sites or rates of change within PAs with those in areas immediately outside them may yield biased estimates of the effectiveness of protection. PAs tend to be located in remote, inaccessible areas that are inherently less likely to be converted [13], [26]. Consequently, comparing rates of change within PAs with those in randomly selected control sites that may be inherently more vulnerable to change (for reasons not attributable simply to their lack of formal protection, e.g. remoteness, distance from roads) may inflate the apparent effectiveness of protection (e.g. [10]). Matching PAs to unprotected sites with similar characteristics, however unrepresentative both might be of the wider landscape, allows an assessment of PA effectiveness that controls for confounding effects that might independently drive the likelihood both of PA designation and of conversion [12]. Such matched studies typically report lower estimates of PA effectiveness relative to unmatched estimates [10], [12], but nevertheless conclude that PAs are generally effective at reducing loss of natural habitats [10], [12], [26]. Estimates of the effectiveness of PAs are further complicated by “leakage” (e.g. [12], [27]), whereby land-cover change is reduced within a PA only for it to be displaced to adjacent areas. Thus, comparisons of rates of change within a PA with rates in the immediately surrounding area will exaggerate the apparent benefits of protection. Leakage might accelerate the rate at which PAs become isolated fragments of natural habitat in otherwise denuded or depauperate landscapes [27].

We quantified the extent to which protection reduces the rate of loss of natural land-cover in sites of recognised biodiversity value using a dedicated land-cover change assessment tool which uses remotely-sensed data at a 30-m resolution - a suitable scale for measuring land-cover change [28]. Change across a 20-year period was assessed within and around a large number of Important Bird Areas (IBAs) in Africa. IBAs are sites of global significance for the conservation of the world’s birds, identified using semi-quantitative criteria [29], and are Key Biodiversity Areas [30]. The most prevalent threats to African IBAs are associated with land-cover change [31]. Through matching, we identified a set of sample points with similar characteristics from comparable protected and unprotected IBAs. This enabled us to quantify rates of land-cover change within and around protected and unprotected IBAs. The effect of protection on rates of loss of natural land-cover was assessed using a survival analysis.

## Methods

### Site selection and matching

Protected and unprotected IBAs were identified by intersecting the digitised boundaries of the 793 IBAs in sub-Saharan Africa and Madagascar (from BirdLife International’s World Bird Database [WBDB]) with the boundaries of all 1580 nationally designated African PAs (from the World Database of Protected Areas [IUCN categories I to VI]) that had digitised boundaries [32]. IBAs that fell wholly or predominantly (>90%) within the boundaries of pre-1985 PAs were classed as protected, while those that did not overlap any part of a PA (whether designated before or after 1985) were classed as unprotected. Exclusion of areas designated after 1985 ensured that sites whose status changed immediately before or during the assessment period were not included in the analysis. IBAs that partially overlapped (<90%) a PA (irrespective of designation date) were excluded. Additionally, IBAs smaller than 10 km^2^ were excluded as the small sample sizes obtained from the sampling protocol might have resulted in a non-representative assessment of land-cover. Of the remaining 692 IBAs, 509 were classed as protected and 183 as unprotected.

Matching was done in two stages. First we matched at the level of whole sites to identify a set of generally similar protected and unprotected IBAs. Second, after assessing land-cover at sample points across these sites (see “***Land-cover change assessment”*** below), we applied a more stringent matching process at the level of individual sample points to identify a subset of these points that were very closely matched between protected and unprotected IBAs. This was done using variables expected to influence the likelihood of land-cover change (e.g. [12], [26]).

Matching was done using the MatchIt package [33] in *R* [34]. For the initial site-level matching, protected IBAs were matched with unprotected IBAs. We used the command ‘subclass’ to assign all 692 IBAs to one of 150 groups, meaning an unprotected IBA could be matched with more than one protected IBA and vice versa. Matching was based on IBA area, mean altitude (both sourced from WBDB), mean distance from roads (www.mapability.com/info/vmap0_intro.html), mean human population density (5-km data; [35]) and the most extensive GLC2000 land-cover class in the IBA (from 1-km data; [36]). This resulted in the selection of 45 protected and 48 unprotected IBAs (Figure S1, Table S1). For these, we assessed land-cover three times over a c. 20-year period at a large number of sample points spaced regularly across the IBA and a surrounding 20-km wide buffer (see “***Land-cover change assessment”*** below). We then sub-sampled these points to produce a set of matched points from within the protected and unprotected IBAs. We used 0.5 SD calipers [10] in MatchIt [33], which set a maximum acceptable difference between matched points (in this case 0.5 standard deviations for each matching covariate). This increases the quality of the matches, at the expense of reducing the number of matched points. For the matching, we again used altitude, human population, distance from roads and land-cover (from the first visual assessment rather than GLC2000), but points were also matched with respect to their occurrence in an IBA or a 20-km buffer around an IBA. The matching selected 14,245 sample points for protected IBAs and the same number for unprotected IBAs, such that the matching variables were very similar across these two groups (Table S2). These matched points were then used in subsequent statistical assessments of protected area effectiveness.

### Land-cover change assessment

We used a grid-point sampling protocol [37] to assess land-cover change [38]. Satellite images were used to identify dominant land-cover within 300×300-m sample boxes (‘points’) distributed across each IBA and a surrounding 20-km buffer. Points were spaced 1.5 km apart within IBAs (0.5 km apart in IBAs <50 km^2^) and 3 km apart in their buffers (Figure S2). Visual interpretation of land-cover from satellite images from three time periods (1981–1994, 1995–2004 and 2005–2009) was undertaken for each point using a specially designed graphical user interface (GUI), now available as a web tool (http://landcover-change.jrc.ec.europa.eu; [38]). Images from each time period were georeferenced to each other and displayed simultaneously to allow direct comparison. Land-cover in 1981–2002 was assessed from Landsat images obtained from www.landcover.org as part of Geocover [39]. Land-cover in 2003–2009 was assessed either from Landsat images (from http://glovis.usgs.gov) or purchased Aster images. All images had a spatial resolution of 30 m. Points for which no data were available (due to cloud, poor image quality or the failure of Landsat 7’s scan line corrector after 2003) in one or more of the sampling periods were excluded. A small number of points in the buffers of unprotected IBAs that overlapped nearby PAs were excluded.

Land-cover in each of the three time periods was allocated to one of 11 broad categories (based on the Land-cover Classification System (LCCS); [40]). These were: closed forest; open forest; mosaic of natural and agricultural vegetation; shrub; herbaceous; tree and shrub crops; arable agriculture; open water; flooded shrub and herbaceous; urban; and bare (Table S3). In order to standardise interpretation, guidelines were prepared in advance containing examples of each land-cover category. Eight IBAs were interpreted by both analysts (AEB and GWE). There were no significant differences between analysts in their estimates of the proportion of any of the land-cover categories on these IBAs in either initial (χ^2^ = 0.35, *P* = 0.6) or final (χ^2^ = 0.05, *P* = 0.8) land-cover assessments. Analysts were unaware at the time of analysis of the protection status of the IBA or whether individual points were in IBAs or in buffers.

### Accuracy

The accuracy of interpretations was evaluated by comparison with very high resolution images from Google Earth at 20 IBAs. Nine of these IBAs were dominated by closed or open forest, three by shrub, three by arable agriculture, two by herbaceous, one by a mosaic of natural and agricultural vegetation, one by bare and one by open water. Forty sample points were selected at random from each of these 20 IBAs (including buffer zones) and the land-cover shown in Google Earth images (dating from 2006–2009) was compared with land-cover identified from the Landsat/Aster images for the 2005–2009 time period. Grouping of land-cover categories into non-natural cover (mosaic of natural and agricultural vegetation; tree and shrub crops; arable agriculture; urban) and natural cover (all other categories) gave an overall interpretation accuracy of 93.9% (±0.008). Accuracy was 87.8% (±1.48) when split into five broad categories of forest (closed forest and open forest), shrub/herbaceous/flooded (shrub; herbaceous; flooded shrub and herbaceous) agricultural (mosaic of natural and agricultural vegetation; tree and shrub crops; arable agriculture; urban), open water, and bare).

### Statistical analysis

We modelled the loss rate of natural land-cover at each of the matched points that held natural land-cover in the initial year of assessment using a generalised linear mixed model. Retention (0) or conversion (1) of natural land-cover was modelled as a binary dependent variable, with the number of years between observations (i.e. exposure time) fitted as a binomial denominator. This accounted for differences in the time interval between assessments and expressed the dependent variable as an estimate of annual rate of conversion that was comparable between sites. Conversion was defined as either the loss of open or closed forest to any other land-cover type or replacement of any of the other natural land-cover types (shrub, herbaceous, flooded shrub and herbaceous, open water, or bare) with non-natural (agricultural/natural mosaic, tree crops or urban.) If the natural land-cover was retained throughout, the number of years between the first and third assessments was taken as the exposure time. If land-cover changed between the initial and second assessments or between the second and final, we assumed that conversion had occurred midway through the intervening period and calculated the exposure time accordingly. None of the points converted between the initial and second assessments had returned to their original state by the final assessment.

Models were fitted with PROC GLIMMIX in SAS, specifying a binomial error distribution. The Laplace approximation was specified to allow comparison of model fit using AIC values between mixed-effects models containing different sets of fixed factors [41]. The binary land-cover change variable was modelled as a function of a 4-level protection class (within a protected IBA; within a 20-km buffer around a protected IBA; within an unprotected IBA; within a 20-km buffer around an unprotected IBA), distance of the point to the IBA boundary (or, where they differed, to the boundary of the PA), and the interaction between class and distance to boundary. The identity of the IBA, nested within country, was fitted as a random effect to account for the potential non-independence of points associated with the same IBA and for possible systematic differences in rates of land-cover change between countries. Thus, the model took the form of: n/t =  class+distance+class*distance +IBA(country), where n is either 1 (converted) or 0 (retained), and t is exposure time (in years).

## Results

### Matching protocol

The sets of protected and unprotected IBA points identified by the matching process were well balanced, with similar distributions of the matching covariates in the two groups (Table S1). Whilst mean altitude and human population were higher across the protected points, the differences in the empirical cumulative distribution function means were zero or close to zero for all variables, indicating a good match.

### Land-cover change

Across all of the matched points, rates of forest loss were greater than rates of loss of shrub, herbaceous and flooded land-cover. Forest cover remained intact on only 81.79% of originally forested points, compared to 91.76% for shrub, herbaceous and flooded cover (which formed the majority of all points) (Table 1). The extent of conversion to agriculture, mosaic or urban among both these types of points was similar at around 5 – 6% from first to last assessment, but around 13% of originally forested points were converted to shrub, herbaceous or flooded cover by the final assessment (Table 1).

**Table 1:** Land-cover categories of points included in the study, expressed as % of points in the final assessment relative to the initial assessment.

	Final assessment					
Initial assessment	Shrub/herbaceous/flooded	Forest	Water	Bare	Agriculture/mosaic/urban	Sample size
Shrub/herbaceous/flooded	91.76	1.28	0.25	0.12	6.59	20919
Forest	13.04	81.79	0.03	0.07	5.06	5926
Water	4.29	0.33	94.00	1.06	0.33	1516
Bare	12.40	0.78	5.43	79.84	1.55	129

Only points with natural or semi-natural land-cover in the first assessment were included in the analyses. See Table S1 for definitions of land-cover types.

The parameter estimates from the null model indicated that the annual rate of loss of natural land-cover across all points was 0.34%±0.004 per year (a model for shrub and herbaceous will be similar to all land-cover, given the dominance these vegetation types in the dataset.) The models indicated that the annual rate of forest loss alone was over twice that of all natural land-cover (0.75%±0.009). Across all land-cover types the model with greatest support contained protection class (the 4-level factor), distance to PA boundary and their interaction term (Table 2), as well as the random effect for IBA nested within country. Inclusion of the protection class factor resulted in the greatest reduction in AUC in the model.

**Table 2:** Summary of fit of models (ranked by **Δ**AIC) to loss of natural land-cover on IBAs in Africa at points matched with calipers, modelled as a function of class (a 4-level factor: within a protected IBA, within a 20-km buffer around a protected IBA, within an unprotected IBA, or within a 20-km buffer around an unprotected IBA), distance to IBA boundary (or, where they differed, to the boundary of the PA), and the interaction between class and distance to boundary.

Model	AIC	ΔAIC
Class + Distance +Class*Distance	19480.52	0
Class + Distance	19498.60	18.08
Class	19503.37	22.85
Distance	19749.94	269.42
**(Null model**	**19760.44**	**279.92)**

All possible models and the null model are shown.

**Figure 1:**
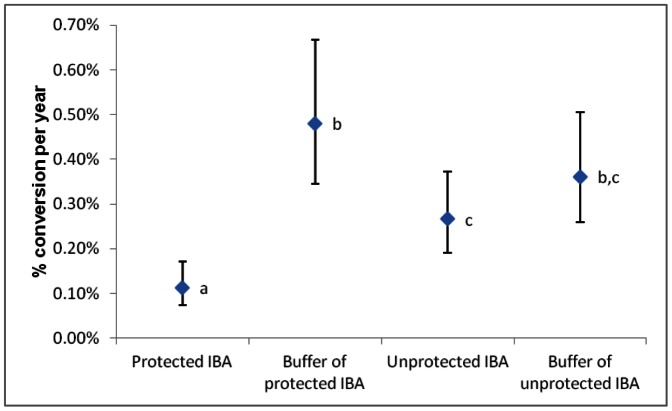
Conversion rates for all natural land-cover (based on parameter estimates with 95% CL) for classes of points estimated from the model with the strongest support (Table 2). Point classes sharing the same letters did not differ significantly from each other.

**Figure 2:**
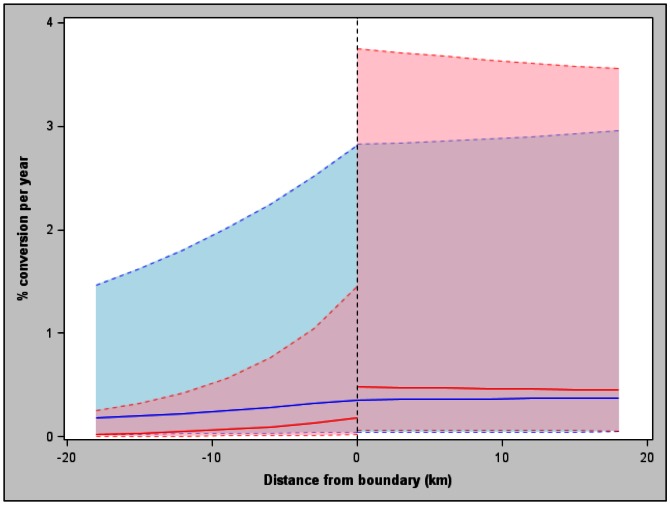
Modelled relationship (solid lines) with 95% CI (dashed lines) between conversion rate of all natural land-cover inside and outside protected IBAs (red) and inside and outside unprotected IBAs (blue). For ease of interpretation, distances inside the IBA boundary (in km) are shown as negative values.

Rates of loss of natural land-cover were 58% lower inside protected IBAs than inside unprotected IBAs (Figure 1). Rates of loss of natural land-cover were 67% lower inside protected IBAs than in their immediately surrounding buffer zones, but did not differ greatly between those in unprotected IBAs and their buffers (Figure 1). Rates of land-cover change were lowest in the centre of both protected and unprotected IBAs, and increased toward site boundaries (Figure 2).The effect of protection was very similar, with rates of forest loss on protected IBAs being 43% of those on unprotected IBAs (Figure S3).

## Discussion

Annual rates of loss of natural land-cover in protected IBAs were less than half of those in IBAs with no legal protection. Estimates of annual rates of loss of all natural land-cover types (0.34%) and of forest alone (0.75%) were similar to estimates of 0.3% and 0.7% respectively, previously reported across sub-Saharan Africa between 1975 and 2000 [37], suggesting both that our approach is robust and that our sample is representative of the continent, despite the oversampling in our study of protected areas and of priority sites for biodiversity conservation.

Our finding that protection is effective at reducing land-cover change on sites of conservation importance supports previous studies that have suggested that site protection has a positive effect on natural land-cover across PAs in general ((e.g. [8], [10], [12], [42]) although this result is not universally reported [43], [44]), and our use of matching reduces the potential for confounding effects to bias results. Studies that have assessed PA effectiveness using matching are perhaps the most reliable available, as assessments that do not involve matching have been shown to overestimate the effectiveness of PAs [10], [13]. The similarity in the relative differences in rates of loss of forest and of all natural vegetation types on protected and unprotected IBAs indicates that studies that have considered forest loss alone might be representative of the relative effectiveness of protection for all natural land-cover types. However, the differences in absolute rates of loss between protected and unprotected sites were almost twice as high for forest as for other land-cover types, suggesting that these studies should not be used to indicate the absolute benefits of protection for all natural habitats.

Rates of loss of natural land-cover around protected IBAs in our sample were far higher than those within protected IBAs. This indicates that protection reduces the rate of land-cover change, but it is not possible to assess whether or not this is also evidence of leakage without knowledge of background rates of conversion in the landscapes in which protected IBAs and their immediate surroundings lie. There were no differences in loss rates in the buffers around protected and unprotected IBAs, providing no consistent evidence of leakage. However, the wide error bars around the parameter estimates for the buffer areas indicate considerable variation between IBAs and, potentially, a lack of power to detect significant leakage effects in the analysis.

Conversion within protected IBAs decreased away from the site boundary, but the rate of change outside protected IBAs did not vary with distance from boundary. This does not follow the theoretical model of leakage suggested by Ewers and Rodrigues [27]. Conversion also decreased with distance from boundary inside unprotected IBAs. This might indicate conversion at the edge of protected and unprotected IBAs is a consequence of landscape configuration, rather than relating to protected area management. Some previous studies (which did not consider potential confounding effects) have presented evidence of leakage [45], [46], but others have found none [12] and some even discerned a positive leakage of conservation benefits into surrounding areas [8]. This topic needs further study, due to the potential for leakage to produce isolated patches of natural habitat in PAs [27]. Analysis of changes in land-cover at matched points along transects across protected and unprotected IBAs, extending beyond the 20-km limit we used here, may be informative.

Our data on land-cover change were collected using a tool designed for the rapid visual analysis of freely available satellite images, which provided a cheap, simple monitoring tool that might be useful in other situations in which resources for field assessment are limited. It took one to two working days to assess long-term land-cover change across an IBA and its buffer. However, we were often unable to differentiate some categories of land-cover (e.g. open vs. closed forest or shrub vs. herbaceous cover). Therefore, only extensive or less subtle changes are likely to be identified. Despite this, our approach could fill gaps in field-based monitoring [47], and could be complemented in the future by other methods for measuring tropical forest and shrubland degradation [48], [49] or by indirect measurements of land-cover dynamics, such as fire detection [50].

In summary, protection is effective at reducing (though not stopping) loss of natural land-cover within sites of high conservation importance. This conclusion is based on changes in all natural land-cover types (unlike many previous studies which consider only forest), at sites of defined conservation importance. Our results suggest that the proposed expansion of the global terrestrial protected area network [4], especially if appropriately targeted [5], will be an effective means to reduce habitat loss and so conserve biodiversity. We found no evidence that such expansion will necessarily result in increased pressures on natural land-cover in areas surrounding protected areas through leakage, but this topic needs further, dedicated study.

## Supporting Information

Figure S1 **Distribution of all African IBAs (stars), showing the 93 IBAs covered in the assessment of land-cover change (circles).** Blue: protected IBAs, red: unprotected IBAs.
(DOCX)

Figure S2 **Example of distribution of sample points within an IBA (shaded area) and in a 20-km buffer around it.** Points within the IBA are 1.5 km apart, those in the buffer are 3 km apart, reflecting the higher density of sampling points used inside IBAs.
(DOCX)

Figure S3 **Conversion rates of forest alone (based on parameter estimates from model with 95% CL) for classes of points estimated from the model with the strongest support (** **Table 2** **).** Point classes sharing the same letters did not differ significantly from each other.
(DOCX)

Table S1 **IBAs selected for land-cover change assessment based on site-level matching, with characteristics used in this site-level matching.**
(DOCX)

Table S2 **Summary of and comparison between matching covariates across 28,490 points split equally between those with and without protection, following matching in MatchIt using calipers  = 0.5 SD.** Land-cover variables were identified from visual assessment. In addition to the basic statistics, the differences in the empirical cumulative distance function are also given, for which values closer to 0 indicate better matching.
(DOCX)

Table S3 **Descriptions of land-cover classes used in visual interpretation, based on Land Cover Classification System (LCCS).**
(DOCX)
