# Supplementary material for: Protection Reduces Loss of Natural Land-Cover at Sites of Conservation Importance across Africa
Source: PLoS One. 2013 May 29;8(5):e65370. doi: 10.1371/journal.pone.0065370 (PMC3667134; doi:10.1371/journal.pone.0065370)
Supplement: Figure S1 — Distribution of all African IBAs (stars), showing the 93 IBAs covered in the assessment of land-cover change (circles). Blue: protected IBAs, red: unprotected IBAs. (DOCX) [file pone.0065370.s001.docx]

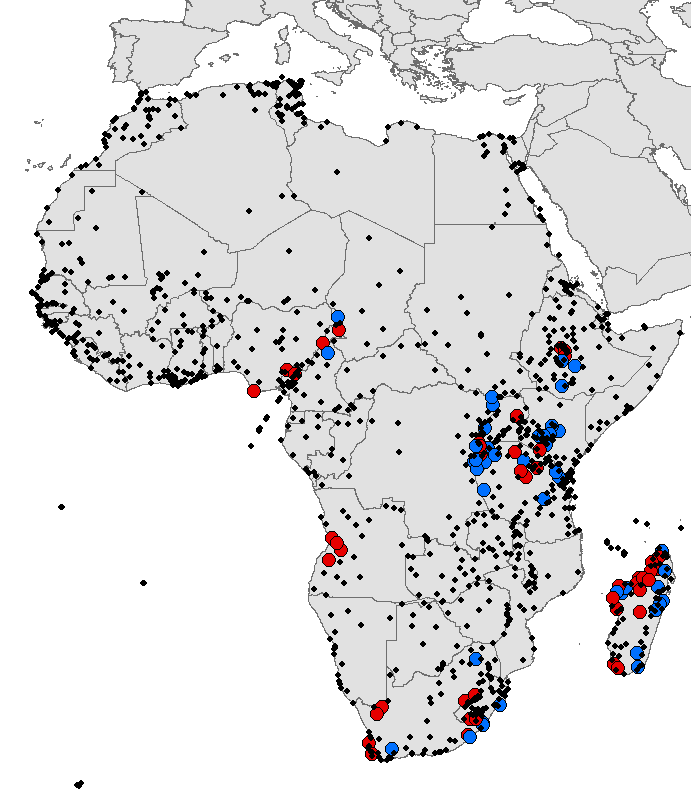


Figure S1. Distribution of all African IBAs (stars), showing the 93 IBAs covered in the assessment of land-cover change (circles). Blue: protected IBAs, red: unprotected IBAs.
