# Supplementary material for: Protection Reduces Loss of Natural Land-Cover at Sites of Conservation Importance across Africa
Source: PLoS One. 2013 May 29;8(5):e65370. doi: 10.1371/journal.pone.0065370 (PMC3667134; doi:10.1371/journal.pone.0065370)
Supplement: Figure S2 — Example of distribution of sample points within an IBA (shaded area) and in a 20-km buffer around it. Points within the IBA are 1.5 km apart, those in the buffer are 3 km apart, reflecting the higher density of sampling points used inside IBAs. (DOCX) [file pone.0065370.s002.docx]

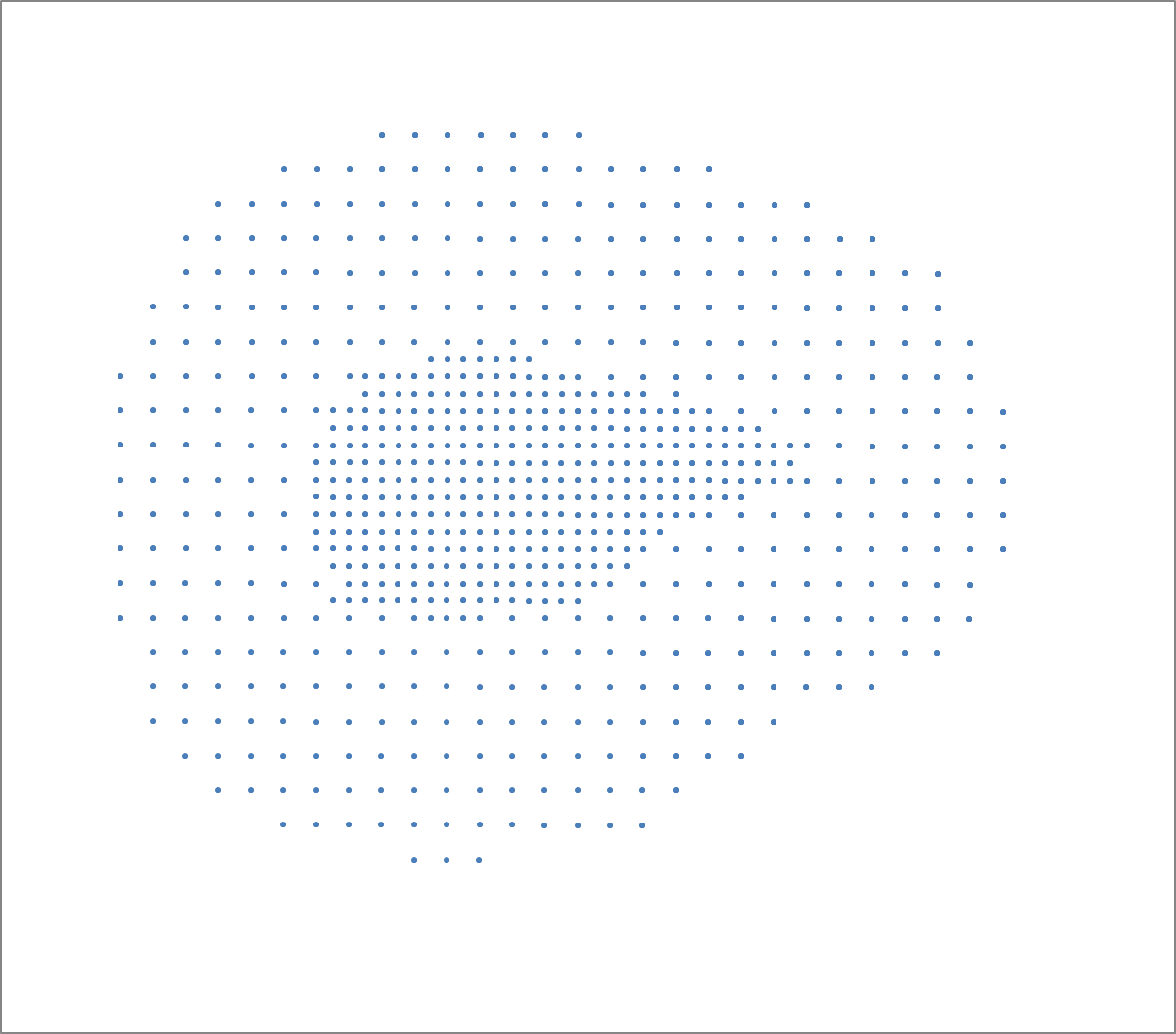


Figure S2. Example of distribution of sample points within an IBA (shaded area) and in a 20-km buffer around it. Points within the IBA are 1.5 km apart, those in the buffer are 3 km apart, reflecting the higher density of sampling points used inside IBAs.
