# Supplementary material for: Protection Reduces Loss of Natural Land-Cover at Sites of Conservation Importance across Africa
Source: PLoS One. 2013 May 29;8(5):e65370. doi: 10.1371/journal.pone.0065370 (PMC3667134; doi:10.1371/journal.pone.0065370)
Supplement: Figure S3 — Conversion rates of forest alone (based on parameter estimates from model with 95% CL) for classes of points estimated from the model with the strongest support ( Table 2 ). Point classes sharing the same letters did not differ significantly from each other. (DOCX) [file pone.0065370.s003.docx]

Figure S3. Conversion rates of forest alone (based on parameter estimates from model with 95% CL) for classes of points estimated from the model with the strongest support (Table 2). Point classes sharing the same letters did not differ significantly from each other.
