# Supplementary material for: Protection Reduces Loss of Natural Land-Cover at Sites of Conservation Importance across Africa
Source: PLoS One. 2013 May 29;8(5):e65370. doi: 10.1371/journal.pone.0065370 (PMC3667134; doi:10.1371/journal.pone.0065370)
Supplement: Table S1 — IBAs selected for land-cover change assessment based on site-level matching, with characteristics used in this site-level matching. (DOCX) [file pone.0065370.s004.docx]

Table S1. IBAs selected for land-cover change assessment based on site-level matching, with characteristics used in this site-level matching.

| IBA | Protect area | Area (ha) | Mean altitude (m) | Mean dist  to road (km) | Human population density(per km) | Dominant land cover  (GLC2000) |
| --- | --- | --- | --- | --- | --- | --- |
| AO007 | No | 2000 | 912.69 | 3 | 20.91 | forest |
| AO011 | No | 5000 | 763.48 | 2 | 15.21 | forest |
| AO018 | No | 2000 | 1491.87 | 5 | 15.21 | forest |
| AO019 | No | 6000 | 1800.04 | 4 | 58.52 | forest |
| BI002 | Yes | 37870 | 2229.58 | 13 | 220.35 | forest |
| BI003 | Yes | 43630 | 1472.04 | 19 | 117.54 | agriculture |
| BI004 | Yes | 9000 | 668.41 | 7 | 171.22 | agriculture |
| BI005 | Yes | 3300 | 1854.36 | 2 | 148.49 | forest |
| CM001 | Yes | 4500 | 292.88 | 1 | 46.15 | agriculture |
| CM004 | No | 50000 | 306.43 | 2 | 81.03 | water |
| CM006 | No | 5000 | 283.69 | 3 | 52.66 | agriculture |
| CM007 | Yes | 180000 | 368.75 | 6 | 7.16 | shrub |
| CM013 | No | 1000 | 1947.08 | 5 | 84.76 | mosaics |
| ET030 | No | 9000 | 2087.1 | 2 | 151.95 | agriculture |
| ET038 | No | 42580 | 2255.44 | 15 | 174 | agriculture |
| ET041 | No | 65400 | 1640.52 | 8 | 111.19 | water |
| ET048 | Yes | 88700 | 1666.74 | 11 | 172.53 | agriculture |
| ET054 | Yes | 247000 | 3505.96 | 13 | 57.92 | agriculture |
| ET064 | Yes | 250000 | 1646.16 | 6 | 12.19 | grass |
| KE001 | Yes | 179900 | 2852.67 | 7 | 55.21 | forest |
| KE005 | Yes | 271000 | 2713.46 | 14 | 48.07 | forest |
| KE031 | Yes | 87000 | 559.4 | 5 | 5.12 | grass |
| KE033 | Yes | 29600 | 932.44 | 3 | 15.31 | grass |
| KE036 | Yes | 11700 | 1645.1 | 3 | 219.13 | grass |
| KE046 | No | 7200 | 1820.94 | 2 | 58.36 | water |
| KE047 | No | 10500 | 617.89 | 3 | 3.86 | water |
| KE049 | Yes | 18800 | 1844.02 | 4 | 52.03 | grass |
| MG003 | Yes | 20030 | 850.55 | 8 | 11.91 | forest |
| MG006 | Yes | 18225 | 212.01 | 3 | 15.97 | forest |
| MG009 | No | 61220 | 0 | 4 | 38.41 | forest |
| MG012 | No | 163100 | 93.14 | 21 | 9.33 | forest |
| MG015 | Yes | 32090 | 1082.84 | 7 | 66.94 | forest |
| MG019 | No | 11490 | 1205.85 | 4 | 21.4 | mosaics |
| MG020 | No | 60700 | 56.74 | 6 | 15.03 | water |
| MG021 | No | 104800 | 101.8 | 4 | 25.42 | grass |
| MG022 | No | 3750 | 0 | 7 | 6.75 | forest |
| MG023 | No | 180000 | 0 | 13 | 9.7 | forest |
| MG024 | No | 148200 | 0 | 5 | 17.58 | shrub |
| MG028 | No | 90110 | 0 | 20 | 3.53 | forest |
| MG029 | Yes | 42200 | 1217.49 | 6 | 9.35 | forest |
| MG030 | Yes | 21742 | 136.11 | 8 | 4.59 | shrub |
| MG031 | No | 10000 | 26.86 | 3 | 20.88 | grass |
| MG032 | Yes | 7900 | 207.2 | 11 | 2.43 | shrub |
| MG033 | Yes | 12080 | 65.67 | 12 | 4.66 | forest |
| MG035 | No | 8300 | 0 | 8 | 2.77 | forest |
| MG038 | No | 41500 | 2.71 | 4 | 4.41 | forest |
| MG048 | Yes | 2228 | 396.76 | 8 | 54.06 | mosaics |
| MG049 | Yes | 11900 | 905.08 | 17 | 16.48 | forest |
| MG054 | Yes | 10685 | 1022.72 | 9 | 13.75 | forest |
| MG058 | No | 3500 | 1278.22 | 3 | 132.63 | water |
| MG063 | No | 98000 | 262.39 | 11 | 3.91 | shrub |
| MG070 | No | 17800 | 148.63 | 17 | 13.55 | shrub |
| MG073 | Yes | 12420 | 332.27 | 9 | 46.1 | shrub |
| MG075 | No | 114700 | 136.28 | 11 | 18.19 | shrub |
| MG083 | Yes | 28250 | 1118.87 | 29 | 5.72 | forest |
| NA012 | No | 2100 | 8.64 | 1 | 0.07 | bare |
| NG001 | No | 72000 | 511.45 | 6 | 66.43 | forest |
| NG017 | No | 7900 | 183.04 | 45 | 167.08 | forest |
| RW001 | No | 8500 | 2088.53 | 11 | 456.17 | agriculture |
| RW002 | Yes | 15000 | 3108.19 | 7 | 418.05 | agriculture |
| RW003 | Yes | 100000 | 1349.44 | 22 | 45.78 | agriculture |
| RW005 | No | 30000 | 1393.41 | 16 | 345 | agriculture |
| TZ005 | Yes | 323000 | 1277.95 | 17 | 47.75 | forest |
| TZ014 | Yes | 350000 | 1340.13 | 4 | 51.26 | forest |
| TZ015 | Yes | 220000 | 1470.86 | 55 | 41.2 | agriculture |
| TZ020 | No | 4000 | 1063.77 | 2 | 61.57 | grass |
| TZ026 | No | 12000 | 1036.06 | 21 | 48.39 | water |
| TZ034 | No | 1100 | 1537.08 | 2 | 40.91 | grass |
| TZ042 | No | 30000 | 1136.59 | 4 | 102.5 | water |
| TZ063 | Yes | 25000 | 1562.36 | 5 | 36.65 | forest |
| TZ064 | Yes | 62861 | 2035.09 | 32 | 45.04 | forest |
| TZ071 | Yes | 38169 | 1489.74 | 8 | 95.33 | forest |
| UG001 | Yes | 4750 | 2576.57 | 7 | 265.15 | forest |
| UG003 | No | 5100 | 2018.92 | 2 | 249.95 | agriculture |
| UG006 | Yes | 76600 | 1252.62 | 6 | 109.38 | forest |
| UG021 | Yes | 15800 | 700.38 | 2 | 18.71 | forest |
| UG022 | Yes | 38400 | 908.05 | 11 | 19.19 | mosaics |
| UG026 | No | 25000 | 1038.68 | 3 | 56.57 | agriculture |
| ZA005 | Yes | 3200 | 1317.13 | 1 | 12.98 | agriculture |
| ZA025 | No | 52000 | 901.21 | 2 | 0.24 | grass |
| ZA027 | No | 45540 | 923.89 | 3 | 0.01 | grass |
| ZA030 | No | 5800 | 1938.2 | 4 | 1.99 | agriculture |
| ZA037 | No | 40000 | 1803.94 | 4 | 9.25 | grass |
| ZA059 | Yes | 1200 | 2.55 | 12 | 36.23 | forest |
| ZA061 | No | 5244 | 1553.41 | 4 | 11.55 | shrub |
| ZA062 | No | 5050 | 1801.51 | 3 | 8.67 | shrub |
| ZA064 | Yes | 1917 | 428.6 | 2 | 19.43 | agriculture |
| ZA065 | Yes | 3257 | 297.29 | 2 | 106.23 | forest |
| ZA067 | No | 2000 | 393.75 | 6 | 36.9 | forest |
| ZA068 | Yes | 6050 | 76.59 | 5 | 24.27 | forest |
| ZA083 | No | 6621 | 26.49 | 2 | 10.9 | grass |
| ZA087 | Yes | 34015 | 943.99 | 9 | 0.21 | grass |
| ZA095 | No | 3000 | 9.5 | 6 | 24.17 | agriculture |
