# Supplementary material for: Protection Reduces Loss of Natural Land-Cover at Sites of Conservation Importance across Africa
Source: PLoS One. 2013 May 29;8(5):e65370. doi: 10.1371/journal.pone.0065370 (PMC3667134; doi:10.1371/journal.pone.0065370)
Supplement: Table S2 — Summary of and comparison between matching covariates across 28,490 points split equally between those with and without protection, following matching in MatchIt using calipers = 0.5 SD. Land-cover variables were identified from visual assessment. In addition to the basic statistics, the differences in the empirical cumulative distance function are also given, for which values closer to 0 indicate better matching. (DOCX) [file pone.0065370.s005.docx]

Table S2. Summary of and comparison between matching covariates across 28,490 points split equally between those with and without protection, following matching in MatchIt [1] using calipers =0.5 SD. Land-cover variables were identified from visual assessment. In addition to the basic statistics, the differences in the empirical cumulative distance function are also given, for which values closer to 0 indicate better matching.

|  | Mean protect | Mean unprot | Std Dev around unprot  mean | Std.Dev for mean difference prot and unprot | Diff.  empirical cumul. dist.  funct.  Median | Diff.  empirical  cumul. dist. funct.  Mean | Diff.  empirical cumul. dist. funct.  Max |
| --- | --- | --- | --- | --- | --- | --- | --- |
| altitude (m) | 1041.33 | 811.29 | 613.29 | 0.27 | 0.05 | 0.08 | 0.22 |
| Dist. to roads (km) | 0.09 | 0.08 | 0.08 | 0.15 | 0.01 | 0.02 | 0.14 |
| Human popn density | 57.87 | 39.44 | 182.08 | 0.07 | 0.06 | 0.08 | 0.24 |
| closed forest | 0.13 | 0.12 | 0.33 | 0.03 | 0.01 | 0.01 | 0.01 |
| open forest | 0.07 | 0.08 | 0.27 | -0.04 | 0.01 | 0.01 | 0.01 |
| shrub | 0.45 | 0.49 | 0.5 | -0.07 | 0.02 | 0.02 | 0.04 |
| herb | 0.27 | 0.23 | 0.42 | 0.09 | 0.02 | 0.02 | 0.04 |
| flooded | 0.02 | 0.02 | 0.15 | -0.02 | 0.00 | 0.00 | 0.00 |
| water | 0.05 | 0.05 | 0.22 | -0.01 | 0.00 | 0.00 | 0.00 |
| bare | 0.00 | 0.00 | 0.07 | -0.01 | 0.00 | 0.00 | 0.00 |

References

1. Ho D, Imai K, King G, Stuart EA (2011) MatchIt: Nonparametric Preprocessing for Parametric Causal Inference. Journal of Statistical Software 42: 1-28.
