# Supplementary material for: Protection Reduces Loss of Natural Land-Cover at Sites of Conservation Importance across Africa
Source: PLoS One. 2013 May 29;8(5):e65370. doi: 10.1371/journal.pone.0065370 (PMC3667134; doi:10.1371/journal.pone.0065370)
Supplement: Table S3 — Descriptions of land-cover classes used in visual interpretation, based on Land Cover Classification System (LCCS). (DOCX) [file pone.0065370.s006.docx]

Table S3: Descriptions of land-cover classes used in visual interpretation, based on Land Cover Classification System (LCCS)[2].

| ***Land cover*** | ***Description*** |
| --- | --- |
| Closed tree cover (>40%) | Closed tree cover, from 100% to 40% canopy cover. Height of the trees greater than 3 metres. Can include trees of all leaf types and phenologies. |
| Open tree cover (40% to 15%) | Open tree cover, with canopy cover from 40% to (20-10)%. Height of the trees greater than 3 metres. Can include trees of all leaf types and phenologies. |
| Mosaic of natural vegetation and agriculture | Cultivated and managed terrestrial area(s) spatially mixed with natural and semi-natural primarily terrestrial vegetation. Class includes proportions of both from 65% to 15%. |
| Shrub cover | Shrub dominated cover (100% to 15%), with possibility of additional layers of closed to open herbaceous (100% to 15%) and/or open tree cover (<15%). Height < 3 m. |
| Herbaceous cover | Herbaceous dominated cover (100% to 4%), with possibility of additional layers of open shrub (<15%). |
| Tree or shrub crops | Plantations or orchards of tree or shrub crops. |
| Arable agriculture | Arable agriculture including small, medium and large field sizes. Can also include fallow fields. |
| Open water | Standing and flowing water. The presence of water can be any combination of permanent, seasonal or temporary. |
| Flooded shrub and herbaceous | Flooded vegetation which can include a thematic mix of shrub, herbaceous and moss or lichen cover. The presence of water can be any combination of permanent, seasonal or temporary. |
| Urban | Areas extensively modified by humans, but not covered by agriculture. Can include built up and non-built up areas, urban vegetated areas, and extraction sites. |
| Bare areas | Percentage vegetation cover of less than 4%. Can include bare soil, bare rock, stony and sandy cover. |

References

2. Di Gregorio A, Jansen LJM (2000) Land Cover Classiﬁcation System (LCCS): Classiﬁcation concepts and user manual. Rome: FAO/UNEP/Cooperazione Italiana.
